# Predicting Risk Propensity Through Player Behavior in DOTA 2: A Cross-Sectional Study

**DOI:** 10.3389/fpsyg.2022.827008

**Published:** 2022-04-29

**Authors:** Sihua Lyu, Nan Zhao, Yichuan Zhang, Wenwen Chen, Haiyan Zhou, Tingshao Zhu

**Affiliations:** ^1^Institute of Psychology, Chinese Academy of Sciences, Beijing, China; ^2^Department of Psychology, University of Chinese Academy of Sciences, Beijing, China; ^3^School of Psychology, Beijing Normal University, Beijing, China

**Keywords:** risk propensity, DOTA 2, machine learning, player behavior, MOBA

## Abstract

As traditional methods such as questionnaires for measuring risk propensity are not applicable in some scenarios, a nonintrusive method that could automatically identify individuals' risk propensity could be valuable. This study utilized Defense of the Ancients 2 (DOTA 2) single match data and historical statistics to train predictive models to identify risk propensity by machine learning methods. Self-reported risk propensity scores from 218 DOTA 2 players were paired with their behavioral metrics. The best-performing model occurred with Gaussian process regression. The root mean square error of this model was 1.10, the correlation between predicted scores and self-reported questionnaire scores was 0.44, the R-squared was 0.17, and the test–retest reliability was 0.67. We discussed how selected behavioral features could contribute to predicting risk propensity and how the approach could be of potential value in the application of perceiving individuals' risk propensities. Moreover, the limitations of our study were discussed, and recommendations were made for future studies in this field.

## Introduction

Risk propensity refers to individuals' tendency to approach or avoid risks in decision-making (Sitkin and Pablo, 1992), which could affect individuals' behaviors in many adversarial and semitransparent situations, including business management (Jaworski and Kohli, 1993), information system management (Huff and Prybutok, 2008), computer hacking (Bachmann, 2010), and even multiplayer online battle arena (MOBA) games. Ferrari (2013) found that League of Legends (LOL) players could confront choices at different risk levels while gaming, hence risk-averse/risk-seeking players would show different behavioral patterns. Thus, in case of confrontation, the ability to perceive opponents' risk propensity could provide some valuable information for inferring opponents' behavioral tendencies and assisting decision-making.

At present, the measurement of risk propensity in psychology is primarily composed of two forms, i.e., lab-based tasks such as decision-making in financial situations and psychological scales containing behavioral statements and questionnaires (Grable and Joo, 1999; Meertens and Lion, 2008; Rubio et al., 2010). Obviously, those traditional methods play an important role in both research and practice. Nevertheless, these measurements of risk propensity highly rely on respondents' willingness. While in the confrontation, it is unlikely to obtain opponents' risk propensity by questionnaires as they are unwilling to expose their characteristics. Therefore, a more convenient and objective measurement to automatically identify individuals' risk propensity can be strongly appealing.

In recent years, many researchers have investigated how to utilize people's online behaviors, such as social networking and gaming behaviors, to evaluate individuals' psychological characteristics (Yee et al., 2011b; Farnadi et al., 2013; Worth and Book, 2015; Majumder et al., 2017; Tandera et al., 2017; Wei et al., 2017). It has been shown that the correlation existed between in-game behaviors and personalities within a certain type of video game. For role-playing games, research shows that players' behavioral traces and linguistic output correlated with their Big Five scores (Griebel, 2006; Yee et al., 2011b). For massively multiplayer online role-playing games, several studies have collected players' in-game behaviors through self-reported questionnaires and searchable databases, investigating the association between different dimensions of personality models with behavioral cues (Yee et al., 2011a; Worth and Book, 2014; Wang and Yu, 2017). For multiplayer online battle arena games and first-person shooter games, researchers reported how the role preference and game actions entangled with players' Big Five personalities (Wang et al., 2019) and other personality traits such as aggression (Delhove and Greitemeyer, 2020). In terms of personality prediction, a number of studies used in-game behaviors to realize personality classification and regression (Bunian et al., 2017; Ammannato and Chiesi, 2020). These predictive models provide new approaches for perceiving individuals' psychological characteristics in a nonintrusive way while traditional measurements are not applicable. In the area of risk propensity, researchers found participants' risk-taking behaviors were positively related to the median recorded distance to the border in a driving computer game (Delgado-Gomez et al., 2021). Stinchcombe et al. (2017) used risky driving behaviors in the simulator as indicators of a richer video game experience. Moreover, Reitter and Grossklags (2019) presented two exploratory studies showing how risk propensity affected in-game behaviors. Ferrari (2013) analyzed how risk-taking/risk-aversive players would react differently during the game. Previous studies have mainly focused on the correlation relationship between in-game behaviors and psychological characteristics, rather than on predictive relationships, especially on risk propensity.

In this study, we intend to build a predictive model and develop a game-based assessment of risk propensity on Defense of the Ancients 2 (DOTA 2). We extracted behavioral features from a single DOTA 2 match and collected historical statistics from OpenDota. Then, we used machine learning algorithms to train models, which could identify players' risk propensity automatically based on these features. Our study provides a new perspective to measure risk propensity and makes up for the shortcomings of traditional measurements.

## Materials and Methods

### Defense of the Ancients 2

Defense of the Ancients 2 is a very popular game, which has been averaging around 450,000 unique online players at any given time each month since October 2021 (Digital Ocean., 2012). With the high popularity of DOTA 2, “The OpenDota Project”^1^ has been developed for parsing DOTA 2 replay files to obtain rich and detailed behavioral information. In addition, OpenDota also provides each player's historical statistics from their matches (Ravari et al., 2020). Thus, DOTA 2 could serve as an ideal platform to acquire players' behavioral data in the game environment, including behavioral details in a certain single match and historical statistics.

### Participants

The correlation coefficients between different psychometric tools usually ranged from 0.39 to 0.68 when measuring the same concept (Craig, 2012). Therefore, to ensure the application value of our model, we expected that the correlation between the self-reported scores and predicted values should exceed 0.4. After the power calculation (alpha level, 0.05, 95% power), at least 63 participants were needed to test whether the correlation coefficient between predicted values and true values was higher than that.

**Figure 1:**
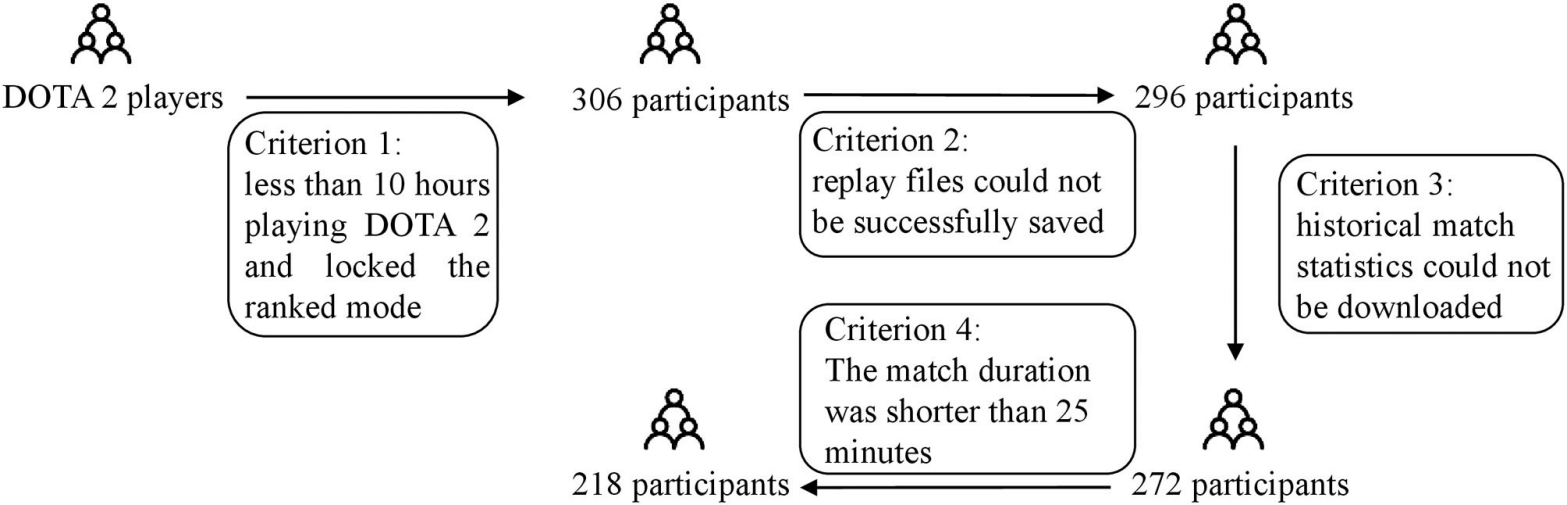
The exclusion criteria of participants' screening process.

To make sure that the players had a basic understanding of the gameplay, we selected subjects who had at least 10 h of playing DOTA 2 or had unlocked the ranked mode. In total, 306 participants were recruited, and 10 participants were removed because their replay files have not been successfully saved. The web scrawler downloaded the majority of participants' historical match statistics on OpenDota (272, 91.9%), but failed on 24 participants. Moreover, a study found that the duration of competitive matches was an indicator of the balance of the matches (Palao et al., 2012), which inspires us to filter out too-short matches to acquire a more reliable dataset. Researchers suggested that the duration for most DOTA 2 games is somewhere between 30 and 40 min (Katona et al., 2019), and another study found that only about 7% of the matches were under 25 min after analyzing 5,744 public replays and 186 professional replays (Hodge et al., 2019). Hence, to ensure that players have taken this match seriously and their opponents are not mismatched, we removed instances less than 25 min from our dataset, and finally, 218 instances were left in the dataset. The complete process and exclusion criteria of participant screening are shown in Figure 1.

Of the 218 valid participants, 209 (95.9%) were men. Participants were aged between 18 and 32, with an average age of 22.98. According to a demographic survey conducted on Reddit^2^, people found that men accounted for about 95% of DOTA 2 players. This is consistent with the gender distribution in this study. For improving the generalization of research findings to a larger population with similar characteristics, it is advised to have representative samples rather than balanced samples in the research (Dickinson et al., 2012).

### Risk Propensity Measurement

We used the Risk Propensity Scale (RPS, Meertens and Lion, 2008) to measure participants' general risk-taking tendencies, rather than domain-specific risk propensity. It includes 7 items and does not have subdimensions. All the statements are rated on a scale ranging from 1 (strongly disagree) to 9 (strongly agree), except for the last item, which is rated on a scale ranging from 1 (risk avoider) to 9 (risk seeker). Meertens and Lion (2008) reported good internal consistency (0.77), adequate test–retest reliability (0.75), and good discriminant validity with other scales in three samples consisting of 522 college students.

### Procedure

In this study, we would briefly introduce the experimental settings and procedure, and more details are available in this study (Lyu et al., 2021).

We posted messages for recruiting participants on MaxPlus, which was a mobile application enabling direct and instant communication between DOTA 2 players. Participants were informed beforehand about all aspects of our study. After we obtained their voluntary consent, participants would finish a survey including basic items related to DOTA 2 play (e.g., “What is your rank in DOTA 2?”), demographic questions such as gender, and the RPS. Before starting the match with bots, participants shared their screens through VooV Meeting, which was a conferencing platform similar to Zoom. Experimenters instructed participants to change default lobby settings as follows: clicking “filling empty slots with bots,” “hard bot difficulty,” and “all pick.” This step of resetting the lobby was to ensure consistent game settings among participants.

To reduce the impact of the experimenter effect on the player's operations, experimenters would end the VooV Meeting after checking the lobby settings. After finishing the match, participants sent their replay files to the experimenters. Finally, a web crawler was used to retrieve historical game metrics from the OpenDota website through participants' Steam ID. Two kinds of historical statistics were not collected. The first type was those items that caused too many missing values such as the participant's win rate of a specifically used hero, as many players might have not played that hero. The second type was items that did not relate to the in-game behavior such as login-to-game location.

Furthermore, to assess the test–retest reliability of our model, 60 participants were randomly picked as the retest group. We followed the same steps above to collect their single match data for the second time and also crawled their historical statistics again later.

### Feature Extraction

After successfully parsing 218 participants' replay files by the OpenDota Project, we acquired the single match data from each participant containing three data tables. The first matches table contained information about team fights (e.g., the number of team fights in the early game), and the second match log table contained information about other teams information except for team fights (e.g., duration of the game). The last player matches table included most of the behavioral data of each player (e.g., the number of towers killed in the game by the player). To build predicting models, feature engineering was necessary (Domingos, 2012). For the single match features, the feature extraction was conducted under the principle proposed by Drachen et al. (2009), which states that we should extract core features that are primarily related to the mechanics of the game. For instance, we calculated time-domain features of each player's killing behavior, such as the mean of hero kills per min, as this behavior is the core of strengthening heroes in DOTA 2. For the historical statistic features, since the features in the historical statistics collected from OpenDota have been well-defined, we could directly use those features. Eventually, we extracted 114 behavioral features from replay files as the single match features, and we crawled 43 historical statistic features, therefore we got 157 features. After filtering features where all values were zero, there were 152 valid behavioral features in total. These in-game behavioral features could be categorized into 3 types:

♦ **Player features**: player features reflect players' performance, such as skills, gold, deaths, and win rate.
♦ **Hero features**: hero features contain two attributes, namely, the hero type and the lane picked by the player at the beginning of the game.
♦ **Match features**: match features include information about team fights and the duration of the game.

### Features Selection

It was expected that there existed some redundant features that would weaken the prediction performance. Hence, features that did not contribute to making predictions were removed. Feature selection was performed through WEKA version 3.8.5, which is a data mining software containing a collection of feature selection tools and prediction models (Witten et al., 2005). In our study, we used the wrapper method as the feature selection technique and adopted cross-validation while evaluating the performance of the subsets. With the selected features, we used a number of machine learning algorithms to train and test the predictive models, through 3-fold cross-validation. Cross-validation is a resampling procedure that could help us reduce the bias in the testing error and model overfitting (Koehrsen, 2018).

## Results

### Descriptive Statistics

Table 1 shows the distribution of 218 participants on ranks, hero types, and competitive positions that they were good at. Over three-quarters of participants have unlocked the ranking mode, and the percentages of participants of each rank were roughly normally distributed. Furthermore, it could be seen that we have covered participants who were good at different hero types and competitive positions.

**Table 1:** Percentage of different player ranks, hero types, and competitive positions.

**Rank**	**Percentage**	**Hero Type**	**Percentage**	**Competitive position**	**Percentage**
None	23.4%	Agility	19.7%	Carry or hard carry	25.7%
Herald	2.3%	Strength	37.2%	Ganker or semi-carry	16.1%
Guardian	5.5%	Intelligence	43.1%	Offlaner	18.8%
Crusader	10.6%			Roamer	19.7%
Archon	15.6%			Babysitter	19.7%
Legend	16.1%				
Ancient	14.7%				
Divine	6.0%				
Immortal	6.0%				

“*None” means players have not unlocked the ranked mode. Players can voluntarily choose whether or not to unlock the ranked mode*.

As for the descriptive statistics of self-reported risk propensity scores, the risk propensity score ranged from 1 to 7.86 among participants, with a mean of 3.21 and a standard deviation of 1.22. The Cronbach's α reliability for the RPS is 0.78.

### The Performance of Regression

As stated above, we used Gaussian process regression (GPR) to evaluate the subset of features for the regression model. After feature selection, the remaining features are shown in Table 2. For a more detailed description of selected features, refer to Supplementary Table 1.

**Table 2:** Remaining features after feature selection.

**Type**	**Features selected**
Single match features	Rune pickups, Skewness of gold per min, Mean of ^a^xp per min, Standard deviation of enemy creep kills per min, Number of attacking items purchasing, Number of comprehensive items purchasing, Times of items using, Skewness of sentry ward planting, Number of necronomicon summoned units kills, Kurtosis of heroes kills per min, Maximum hero hit, Ratio of abilities cast on self, ^b^Ratio of action type 9, ^c^Ratio of action type 10, ^d^Ratio of action type 11, ^e^Ratio of action type 13, ^f^Ratio of action type 14, ^g^Ratio of action type 16, ^h^Ratio of action type 20, ^i^Ratio of action type 23, ^j^Ratio of action type 26, ^k^Ratio of action type 32, ^l^Ratio of action type 36, ^m^Ratio of action type 38, Ratio of damage dealt by player, Ratio of damage dealt to creep, Ratio of damage taken from creep, Mean of observer ward planting per min, Standard deviation of sentry ward planting per min
Historical statistic features	Mean Of Deaths In Recent Matches, Mean Of Xp Per Min In Recent Matches, Mean Of Tower Damage In Recent Matches, Total Number Of Deaths, ^n^KDA, Total Number Of Denies, Lane Efficiency Pct, Total Stun Duration, Total Number Of Comebacks, Loss

*^a^xp means “experience”. ^b−m^A total of 38 different action types parsed from each player's replay. The ratio of a particular action type refers to the percentage of this action type accounted for in combat actions. ^n^KDA is the ratio of the total number of kills plus the total number of assists over the total number of deaths and indicates the player's performance in past matches*.

We used the following machine learning methods support vector regression (SVR), linear regression (LR), GPR, random forest (RF), and bagging to train models based on the selected features. Two best-fitting regression models are shown in Table 3. We can see that the performance of different regression algorithms varied, with GPR having the best performance (RMSE = 1.10, *r* = 0.44, *R*^2^ = 0.17).

**Table 3:** The performance of the regression models with 3-fold cross-validation.

**Method**	**r**	**RMSE**	**R^2^**
GPR	0.44**	1.10	0.17
RF	0.20**	1.20	0.01

“r” is the Pearson's correlation coefficient between predicted values and true values (**p < 0.01), and “RMSE” is the root mean square error. R^2^ is the proportion of the variance in the response variable that can be explained by the predictor variables in the model. GPR, Gaussian process regression; RF, random forest.

**Figure 2:**
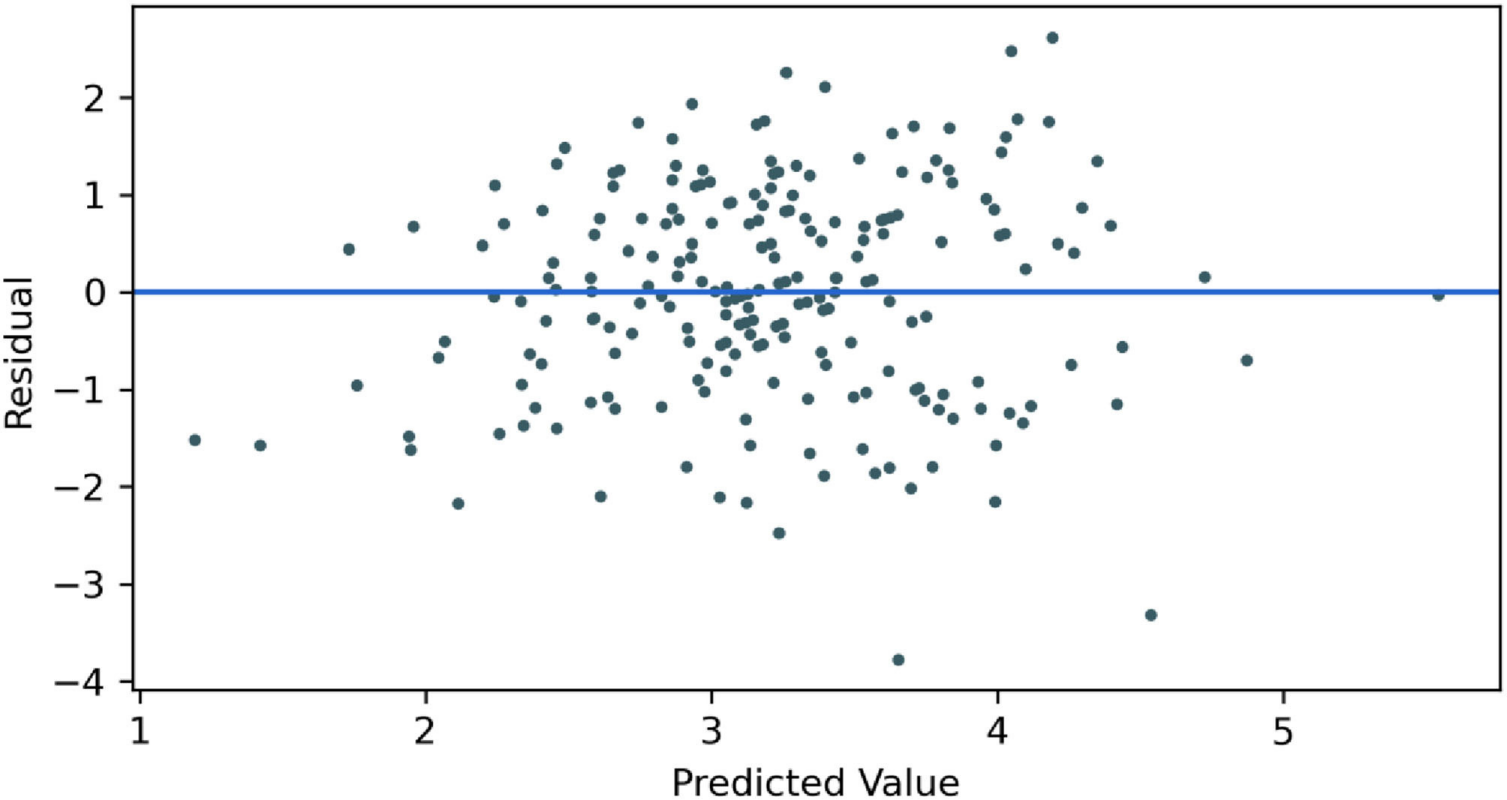
Residuals vs. predictor plot.

We plotted a graph of the GPR model showing the predicted value (x-axis) and the residual (y-axis) in Figure 2. It can be observed that the residuals were symmetrically distributed about the origin, satisfying the independence of residuals. Furthermore, a large proportion of the residuals were distributed between −1 and 1, indicating the reasonable prediction accuracy of the model.

### The Test–Retest Reliability of Regression Models

The best-fitting model did not necessarily perform best in reliability, and thus we calculated the test–retest reliability for all built regression models. First, we removed participants of the test–retest sample from the original dataset, so there was a new training sample consisting of 180 instances remaining for model rebuilding. Second, we adopted the same methods for feature selection and model training as before to build predicting models based on this new training sample. As we have conducted the same experiment twice on the test–retest sample, we would get one set of selected features for each participant from each experiment. Hence, there are two sets of features as inputs for every participant in the test–retest sample to predict their risk propensity. In addition, our rebuilt predictive models could calculate risk propensity scores two times for the same participant, as her/his first and second measurement, based on the two inputs. The correlation coefficient between these two measurements was the test–retest reliability of the model.

Table 4 integrates the test–retest reliability and performance of GPR and RF regression models. Results showed that the test–retest reliability for most of our regression models was more than 0.55, indicating the stability of our models. In addition, the model with the highest test–retest reliability was the GPR model (*r* = 0.67), which also showed the best prediction performance.

**Table 4:** The performance and test–retest reliability of regression models.

	**Method**	**r**	**RMSE**	**R^2^**	**Reliability**
Model1	GPR	0.44**	1.10	0.17	0.67
Model2	RF	0.20**	1.20	0.01	0.56

“r” is the Pearson's correlation coefficient between predicted values and true values (**p < 0.01), and “RMSE” is the root mean square error. R^2^ is the proportion of the variance in the response variable that can be explained by the predictor variables in the model. GPR, Gaussian process regression; RF, random forest.

## Discussion

### The Feasibility of Predicting Risk Propensity by MOBA Game Behavior

We used DOTA 2 behavioral data, including single-match data and historical statistics, and predicting models trained by machine learning to identify players' risk propensity. Several models showed fairly good predictive accuracy as well as test–retest reliability, and GPR outperformed other algorithms in both performance and reliability. Specifically, the root mean squared error was 1.10, the correlation between predicted values and self-reported values was 0.44, the R-squared was 0.17, and the test–retest reliability was 0.67. It possibly signified that GPR was a relatively suitable algorithm for DOTA 2 behavioral datasets.

Some researchers have investigated how to predict players' psychological traits through in-game behavior, but the results did not show strong predictive power (Yee et al., 2011a; Fong and Mar, 2015; Bunian et al., 2017; Wu et al., 2021). For example, Bunian et al. (2017) used hidden Markov models (HMM) to extract the sequence of players' actions and trained classification models for the Big Five personality traits. However, the classification accuracy was not satisfactory, ranging from 54% to 60%. Yee et al. (2011a) attempted to conduct a multiple regression analysis of the Big Five personality traits using behavioral metrics in World of Warcraft. All of the multiple regressions were significant, while the correlation coefficients between predicted values and true values were only between 0.2 and 0.3. In addition, none of these studies have examined the test–retest reliability of the model. We expected that the improvement of correlation between predicted values and true values in this study was related to the extraction of richer behavioral statistics and the selection of algorithms. By exploiting representative behavioral statistics and combining them with a proper learning algorithm, it is possible to improve the accuracy of the predictive model.

Our findings supported that in-game behavior could be utilized to automatically identify players' risk propensity. Players with different levels of risk propensity vary from each other in terms of behavioral patterns in MOBAs. This model enriches the measurement of individuals' risk propensity, and it could be hopefully employed in scenarios where questionnaires and lab-based tasks are inapplicable such as in the confrontation.

### The Features Worked in Predicting Player's Risk Propensity

To further understand how the features in our model contribute to predicting risk propensity, we further analyzed the meaning of the features and grouped them into 3 categories. As the developer of DOTA 2 has not elucidated what features “action types” and two historical statistic features (i.e., lane efficiency of PCT and loss) refer to, such ambiguous features were not within the scope of our discussion.

#### Hero Killing-Related Features

The first category contains features that are associated with the behavior of killing enemy heroes. In DOTA 2, the act of killing enemy heroes itself puts players at a greater risk of being killed, compared with attacking creeps and toppling towers. Therefore, we expected that players' hero killing behavior is correlated with ones' risk propensity. In addition, we further divided the first category into four subcategories based on the meaning of each behavioral feature.

The first subcategory contains the gold/xp-related features: skewness of gold per min, mean of xp per min, and mean of xp per min in recent matches. In DOTA 2, xp is required for leveling up to empower heroes, and gold is the currency used to buy items or revive heroes. Typically, both xp and gold can be earned by killing enemy heroes, enemy nonhero units (creeps or summons), or neutral creeps. However, killing enemy heroes could yield significantly higher gold and xp than killing other behavior each time, although it is riskier and less constant. Therefore, players' patterns of gold and xp acquisition could be strongly influenced by hero killing behavior.

Another subcategory includes features reflecting the damage dealt with or taken from the enemy creep. They are the standard deviation of enemy creep kills per min, the ratio of damage dealt with creep, and the ratio of damage taken from creep. There are two different strategies called farming and active carry in DOTA 2. Farming is a conservative strategy that focuses on enemy creep or neutral creep killing. Active carry is an aggressive strategy that actively engaged in the fighting against enemy heroes (Eggert et al., 2015). Features in this subcategory could reflect the extent to which the player is engaged in killing enemy creep, and thus we can infer the player's tendency between active carries and farming.

The third subgroup consists of features whose values are calculated from hero killing. They are kurtosis of hero kills per min, total stun duration, and KDA. Kurtosis of hero kills per min describes the peakedness of the probability distribution of the number of hero kills per min. Stun is a status effect that is mainly inflicted on enemy heroes, causing a complete lockdown and disabling almost all of the enemy heroes' capabilities. KDA is the ratio of the number of kills plus assists over deaths and indicates the player's performance in the match (Neto et al., 2017; Matsui et al., 2020). The values of these three features are mainly or fully determined by the confrontation between players and opponents.

The number of attacking items purchasing belongs to the last subgroup, as players are more likely to equip themselves with attacking items before launching an attack on enemy heroes.

To further support our categorization statistically, we drew a heatmap to present the correlation coefficients between features in the first category and hero killing behavior (refer to Supplementary Figure 1), and the correlations between the two variables represented by the colored blocks in the heatmap are all statistically significant at 0.01 level. We used the following features to define hero killing behavior: number of hero kills, standard deviation of hero kills per min, kurtosis of hero kills per min, skewness of hero kills per min, number of multi-kills, ratio of damage dealt with heroes, and mean of hero kills per min. Results showed that features in this category are correlated to hero killing behavior to different extents.

#### Information Acquisition-Related Features

The second category contains features that are highly related to information acquisition. They are skewness of sentry ward planting per min, standard deviation of sentry ward planting per min, and mean of observer ward planting per min. Katona et al. (2019) considered that DOTA 2 is a real-time game with hidden information. For example, areas covered in the fog of war are outside of the player's vision, leading that any enemy unit in the fog of war cannot be detected or targeted. Thus, players are supposed to formulate strategies under uncertainty and riskiness in DOTA 2.

Previous literature has shown that individuals with higher risk perception or lower risk propensity are more likely to adopt risk-reducing strategies such as information search before making a decision under uncertainty (Roselius, 1971; Taylor and Dunnette, 1974; Beatty and Smith, 1987; Srinivasan and Ratchford, 1991; Lion and Meertens, 2001; Cases, 2002; Chen and He, 2003; Byzalov and Shachar, 2004; Björk and Kauppinen-Räisänen, 2011). In addition, it has been supported by researchers that risk propensity was negatively correlated with the level of perceived risk (Sitkin and Weingart, 1995; Brockman et al., 2006; Lopez-Nicolas and Molina-Castillo, 2008). Hence, it is reasonable to infer that players with different levels of risk propensity could vary in terms of in-game behavior for information acquisition.

In DOTA 2, sentry ward and observer ward planting are one of the gameplay mechanisms that enable players to acquire extra information. They could grant players vision and enable players to spot the enemy unit that moves by, providing players more information to make less risky decisions.

#### High-Risk Choice-Related Features

Features in the third category are all related to high-risk and high-reward activities. Players' decisions of whether or not to engage in these activities could reflect their risk propensity to some extent.

The first feature is the number of Necronomicon summoned units killed by the player. Necronomicon summoned units (i.e., Necronomicon warrior and Necronomicon archer) can fight for 60 s after being summoned. The Necronomicon warrior can cause the mana loss of players and deal damage at the same time. In addition, it has another ability called *last will* referring that a significant amount of damage will be dealt with the unit that kills the Necronomicon warrior. Moreover, Necronomicon warrior at level 3 is able to detect invisible units, providing a fully unobstructed moving vision. The Necronomicon archer not only removes positive buffs from opponents and slows opponents' movement speed but also increases the movement speed of nearby allied units. Based on the abilities of Necronomicon summoned units, we can conclude that the primary risk for killing summoned units is that the player could suffer a stable mana loss and massive damage, increasing the probability of hero death. As for the rewards, the player who killed these units could gain a very large xp and gold bounty and eliminate the threat brought by the Necronomicon summoned units such as being detected in the moving vision.

The second feature is the total number of comebacks. In DOTA 2, players can choose to revive the hero instantly by costing a large amount of gold when a hero dies. In addition, an additional price for reviving the hero in this way is that the next respawn time for the hero will be extended by 25 s. Hence, we can see that risks for comebacks consist of the huge amount of consumption of gold and the extended respawn time. As for the reward, reviving the hero may help players make a big difference especially when it comes to a critical moment in the game.

The third and fourth features are the average number of deaths in recent matches and the total number of deaths. From the perspective of game design, we can easily reach an agreement that hero death is the major risk that players face whenever they make a strategy in DOTA 2. Hence, the features related to hero death may somewhat reflect the riskiness of the strategy that the player has taken.

Overall, features in this category are all related to riskiness and rewards. In addition, many researchers have suggested that risk-takers focus more on the rewards associated with the risks, while risk avoiders focus more on the costs of the risks (Lopes, 1987; Horvath and Zuckerman, 1993; Anderson and Galinsky, 2006). Thus, risk-takers are more likely to show risk-taking behavior, while risk avoiders are not. This might be contributing to the prediction of risk propensity.

#### Unknown Features

There were still 6 features left that have not been categorized: rune pickups, number of comprehensive items purchasing, times of item used, denies, mean of tower damage per min in recent matches, ratio of abilities cast on self, maximum hero hit, and ratio of damage dealt by the player. We expected that these features might also contribute to risk propensity prediction from other aspects that we currently have not thought of yet. However, in view of the relatively comprehensive elaboration on the features in the three categories, it has shed light on the reason why the GPR model could predict individuals' risk propensity. In addition, for the remaining six features that have not been discussed, future research may provide more insights on that.

### The Possible Implications of Game-Based Assessment

This study demonstrated that it is possible to identify players' risk propensity through their in-game behavior. Games allow us to observe individuals under contexts similar to those in the real world, creating complex scenarios required to evaluate individuals' psychological features such as personality (DiCerbo, 2014). The game-based assessment does not require players to fill in long surveys, and players' match data could be easily approached after their permission. This method opens a new avenue to nonintrusively perceive the player's risk propensity with the low cost through players' behavior in MOBAs.

As our study has investigated how in-game behaviors could be entangled with players' risk propensity, it may also help game companies to customize various strategies for AI systems. Through acquiring any opponent's risk propensity, the AI system may infer his/her following adopted actions. This customization of game mechanics based on players' personalities can improve users' gameplay experience, making games more enjoyable (Nagle et al., 2016; Bourke et al., 2018; Soares et al., 2018).

### Limitations and Future Work

This study has a few limitations. First, our models failed to take players' linguistic output into account while building models. However, players' linguistic output such as communication with teammates is an important part of in-game behavior and may be of great help in identifying players' risk propensity. Second, there might be more attributes that may depict players' in-game behavior, such as frequency domain features of time series data, which have not been investigated. Third, since we only analyzed data acquired from DOTA 2 players, the present predictive models may only be applicable to DOTA 2 players. Moreover, even though we have discussed how features in different categories contribute to predicting risk propensity, we have not investigated the relationship between each feature and risk propensity specifically. Fourth, even though we have found a reasonable level of correlations between the predicted values and true values, the R-squared of models are almost in the range of 1% except for the GPR model. This signifies a proper linear and positive relationship exists between the fitted values and true values for most of our models, and these models can only explain 1% of the variation of the predicted variable. The lower R-squared of other models may be caused by the unsuitability between our dataset and these algorithms. Thus, it probably indicates that in-game behaviors utilized in our study may not be robust predictors of the risk propensity of our recruited sample.

Despite these pitfalls, our results can be still enlightening for future research perceiving risk propensity through in-game behavior. As we have found a medium correlation between fitted values and true values, this may serve as a baseline for future work focusing on exploring and utilizing more attributes such as linguistic output to further improve the performance and reliability of the predicting model. Additionally, future studies could use different types of games as platforms and compare the findings across the games, paying attention to whether the same methods can be applied to other games. If our methods are applicable to other games, the next step could be to investigate the similarity of features in different models and strive to put forward a set of in-game behavior across games that are related to risk propensity.

## Data Availability Statement

The raw data supporting the conclusions of this article will be made available by the authors, without undue reservation.

## Ethics Statement

The studies involving human participants were reviewed and approved by Scientific Research Ethics Committee of the Chinese Academy of Sciences Institute of Psychology. The patients/participants provided their written informed consent to participate in this study.

## Author Contributions

TZ and NZ contributed to the conception, design of the study, and to the final version of the manuscript. YZ and WC were responsible for data collection and the statistical analysis in revising. SL performed the statistical analysis and wrote the manuscript with input from all authors. HZ helped with the manuscript revision. All authors contributed to the article and approved the submitted version.

## Funding

This work was supported by the Key Research Program of the Chinese Academy of Sciences (No. ZDRW-XH-2019-4), the Strategic Priority Research Program of Chinese Academy of Sciences (No. XDC02060300), and the Youth Innovation Promotion Association CAS.

## Conflict of Interest

The authors declare that the research was conducted in the absence of any commercial or financial relationships that could be construed as a potential conflict of interest.

## Publisher's Note

All claims expressed in this article are solely those of the authors and do not necessarily represent those of their affiliated organizations, or those of the publisher, the editors and the reviewers. Any product that may be evaluated in this article, or claim that may be made by its manufacturer, is not guaranteed or endorsed by the publisher.

## Footnotes

^1^ https://github.com/odota

^2^Into The Breach Esports. (2021). r/DOTA 2 Demographic Survey: Results. Retrieved from Reddit: https://docdro.id/ZeJTLar (accessed September 15, 2021).

## References

[B1] Ammannato G. Chiesi F. (2020). Playing with networks: Using video games as a psychological assessment tool. Euro. J. Psychologic. Assess. 36, 973. 10.1027/1015-5759/a000608

[B2] Anderson C. Galinsky A. D. (2006). Power, optimism, and risk-taking. Euro. J. Soc. Psychol. 36, 511–536. 10.1002/ejsp.324

[B3] Bachmann M. (2010). The risk propensity and rationality of computer hackers. Int. J. Cyber Criminol. 4, 643–656.

[B4] Beatty S. E. Smith S. M. (1987). External search effort: an investigation across several product categories. J. Consum. Res. 14, 83–95. 10.1086/209095

[B5] Björk P. Kauppinen-Räisänen H. (2011). The impact of perceived risk on information search: a study of Finnish tourists. Scand. J. Hospital. Tour. 11, 306–323. 10.1080/15022250.2011.593358

[B6] Bourke P. Murphy D. O'Mullane J. Marshall K. Howell S. (2018). “Review of player personality classifications to inform game design,” in 2018 IEEE Games, Entertainment, Media Conference (GEM) (pp. 1–9).

[B7] Brockman B. K. Becherer R. C. Finch J. H. (2006). Influences on an entrepreneur's perceived risk: the role of magnitude, likelihood, and risk propensity. Acad. Entrepreneur. J. 12, 107.

[B8] Bunian S. Canossa A. Colvin R. El-Nasr M. S. (2017). “Modeling individual differences in game behavior using HMM,” in Thirteenth Artificial Intelligence and Interactive Digital Entertainment Conference. Thirteenth *Artificial Intelligence and Interactive Digital Entertainment Conference*. Available online at: https://www.aaai.org/ocs/index.php/AIIDE/AIIDE17/paper/view/15819 (accessed September 8, 2021).

[B9] Byzalov D. Shachar R. (2004). The risk reduction role of advertising. Quantitative Marketing and Economics 2, 283–320. 10.1007/s11129-004-0153-x

[B10] Cases A. S. (2002). Perceived risk and risk-reduction strategies in Internet shopping. Int. Rev. Retail Distribut. Consum. Res. 12, 375–394. 10.1080/09593960210151162

[B11] Chen R. He F. (2003). Examination of brand knowledge, perceived risk and consumers' intention to adopt an online retailer. Total Qual. Manage. Bus. Excell. 14, 677–693. 10.1080/1478336032000053825

[B12] Craig R. J. (2012). “Assessing personality and psychopathology with interviews,” in Handbook of Psychology, Second Edition, eds I. Weiner, J.R. Graham and J.A. Naglieri.7705086

[B13] Delgado-Gomez D. Sujar A. Ardoy-Cuadros J. Moana S. Peñuelas-Calvo I. Aguado D.. (2021). Objective risk assessment using a driving computer game. Euro. Psychiatr. 64, S508–S509. 10.1192/j.eurpsy.2021.1361

[B14] Delhove M. Greitemeyer T. (2020). The relationship between video game character preferences and aggressive and prosocial personality traits. Psychol. Popul. Media 9, 96–104. 10.1037/ppm0000211

[B15] DiCerbo K. E. (2014). Game-based assessment of persistence. J. Educ. Technol. Soc. 17, 17–28.

[B16] Dickinson E. R. Adelson J. L. Owen J. (2012). Gender balance, representativeness, and statistical power in sexuality research using undergraduate student samples. Archiv. Sex. Behav. 41, 325–327. 10.1007/s10508-011-9887-1 22228196

[B17] Digital Ocean. (2012). STEAMCHARTS: An Ongoing Analysis of Steam's Concurrent Players. Available online at: https://steamcharts.com/app/570#48h (accessed March 12, 2022).

[B18] Domingos P. (2012). A few useful things to know about machine learning. Commun. ACM 55, 78–87. 10.1145/2347736.2347755

[B19] Drachen A. Canossa A. Yannakakis G. N. (2009). “Player modeling using self-organization in tomb raider: underworld,” in 2009 IEEE Symposium on Computational Intelligence and Games, pp. 1–8.

[B20] Eggert C. Herrlich M. Smeddinck J. Malaka R. (2015). “Classification of player roles in the team-based multi-player game dota 2,” in International Conference on Entertainment Computing, pp. 112–125.

[B21] Farnadi G. Zoghbi S. Moens M. F. De Cock M. (2013). “How well do your Facebook status updates express your personality?,” in Proceedings of the 22nd edition of the annual Belgian-Dutch conference on machine learning (BENELEARN), p. 88.

[B22] Ferrari S. (2013). “From generative to conventional play: MOBA and league of legends,” in DiGRA Conference, pp. 1–17.

[B23] Fong K. Mar R. A. (2015). What does my avatar say about me? inferring personality from avatars. Personal. Soc. Psychol. Bull. 41, 237–249. 10.1177/0146167214562761 25576173

[B24] Grable J. E. Joo S. H. (1999). Factors related to risk tolerance: a further examination. Consum. Inter. Ann. 45, 53–58.

[B25] Griebel T. (2006). Self-portrayal in a simulated life: projecting personality and values in the sims 2. Game Stud. 6, 601.

[B26] Hodge V. J. Devlin S. M. Sephton N. J. Block F. O. Cowling P. I. Drachen A. (2019). “Win prediction in multi-player esports: Live professional match prediction,” in IEEE Transactions on Games.

[B27] Horvath P. Zuckerman M. (1993). Sensation seeking, risk appraisal, and risky behavior. Personal. Individ. Differen. 14, 41–52. 10.1016/0191-8869(93)90173-Z

[B28] Huff R. A. Prybutok V. R. (2008). Information systems project management decision making: the influence of experience and risk propensity. Project Manage. J. 39, 34–47. 10.1002/pmj.20050

[B29] Jaworski B. J. Kohli A. K. (1993). Market orientation: antecedents and consequences. J. Market. 57, 53–70. 10.1177/002224299305700304

[B30] Katona A. Spick R. Hodge V. J. Demediuk S. Block F. Drachen A.. (2019). “Time to die: death prediction in dota 2 using deep learning. In 2019 IEEE Conference on Games (CoG) (pp. 1-8).

[B31] Koehrsen W. (2018). Overfitting vs. Underfitting: A Complete Example. Towards Data Science.

[B32] Lion R. Meertens R. M. (2001). Seeking information about a risky medicine: effects of risk-taking tendency and accountability. J. Appl. Soc. Psychol. 31, 778–795. 10.1111/j.1559-1816.2001.tb01413.x

[B33] Lopes L. L. (1987). Between hope and fear: the psychology of risk. Adv. Experiment. Soc. Psychol. 20, 255–295. 10.1016/S0065-2601(08)60416-5

[B34] Lopez-Nicolas C. Molina-Castillo F. J. (2008). Customer Knowledge Management and E-commerce: the role of customer perceived risk. Int. J. Inform. Manage. 28, 102–113. 10.1016/j.ijinfomgt.2007.09.001

[B35] Lyu S. Chen W. Zhang Y. Zhu T. (2021). Dark Personality Prediction from Player in-game Behavior: Machine Learning Methods. ChinaXiv:202107.00010. Available online at: http://chinaxiv.org/abs/202107.00010 (Accessed September 15, 2021)

[B36] Majumder N. Poria S. Gelbukh A. Cambria E. (2017). Deep learning-based document modeling for personality detection from text. IEEE Intell. Syst. 32, 74–79. 10.1109/MIS.2017.23

[B37] Matsui A. Sapienza A. Ferrara E. (2020). Does streaming esports affect players' behavior and performance?. Games Cult. 15, 9–31. 10.1177/1555412019838095

[B38] Meertens R. M. Lion R. (2008). Measuring an individual's tendency to take risks: the risk propensity scale. J. Appl. Soc. Psychol. 38, 1506–1520. 10.1111/j.1559-1816.2008.00357.x

[B39] Nagle A. Wolf P. Riener R. (2016). Towards a system of customized video game mechanics based on player personality: relating the Big Five personality traits with difficulty adaptation in a first-person shooter game. Entertain. Comput. 13, 10–24. 10.1016/j.entcom.2016.01.002

[B40] Neto J. A. Yokoyama K. M. Becker K. (2017). “Studying toxic behavior influence and player chat in an online video game,” in Proceedings of the International Conference on Web Intelligence, pp. 26–33.

[B41] Palao J. M. Valades D. Ortega E. (2012). Match duration and number of rallies in men's and women's 2000–2010 FIVB world tour beach volleyball. J. Hum. Kinetics 34, 99. 10.2478/v10078-012-0068-7 23486703 PMC3590823

[B42] Ravari Y. N. Strijbos L. Spronck P. (2020). “Investigating the relation between playing style and national culture,” IEEE Transact. Computat. Intell. AI Games, pp. 1–10.

[B43] Reitter D. Grossklags J. (2019). The positive impact of task familiarity, risk propensity, and need for cognition on observed timing decisions in a security game. Games 10, 49. 10.3390/g10040049

[B44] Roselius T. (1971). Consumer rankings of risk reduction methods. J. Market. 14, 56–61. 10.1177/002224297103500110

[B45] Rubio V. J. Hernández J. M. Santacreu J. (2010). The Objective Assessment of Risk Tendency as a Personality Dimension. Madrid: University Autónoma of Madrid. Available online at: www.uam.es/proyectosinv/psimasd/assessrisk.pdf

[B46] Sitkin S. B. Pablo A. L. (1992). Reconceptualizing the determinants of risk behavior. Acad. Manage. Rev. 17, 9–38. 10.5465/amr.1992.427956415913678

[B47] Sitkin S. B. Weingart L. R. (1995). Determinants of risky decision-making behavior: a test of the mediating role of risk perceptions and propensity. Acad. Manage. J. 38, 1573–1592. 10.2307/256844

[B48] Soares D. L. E. Feijó B. Furtado A. L. (2018). Player behavior and personality modeling for interactive storytelling in games. Entertain. Comput. 28, 32–48. 10.1016/j.entcom.2018.08.003

[B49] Srinivasan N. Ratchford B. T. (1991). An empirical test of a model of external search for automobiles. J. Consum. Res. 18, 233–242. 10.1086/209255

[B50] Stinchcombe A. Kadulina Y. Lemieux C. Aljied R. Gagnon S. (2017). Driving is not a game: Video game experience is associated with risk-taking behaviours in the driving simulator. Comput. Hum. Behav. 69, 415–420. 10.1016/j.chb.2016.12.006

[B51] Tandera T. Suhartono D. Wongso R. Prasetio Y. L. (2017). Personality prediction system from facebook users. Procedia Comput. Sci. 116, 604–611. 10.1016/j.procs.2017.10.016

[B52] Taylor R. N. Dunnette M. D. (1974). Influence of dogmatism, risk-taking propensity, and intelligence on decision-making strategies for a sample of industrial managers. J. Appl. Psychol. 59, 420–423. 10.1037/h0037343

[B53] Wang C. Yu G. (2017). The relationship between player's value systems and their in-game behavior in a massively multiplayer online role-playing game. Game 1, 1404. 10.1155/2017/6531404

[B54] Wang Z. Sapienza A. Culotta A. Ferrara E. (2019). “Personality and behavior in role-based online games,” in 2019 IEEE Conference on Games (CoG), pp. 1–8.

[B55] Wei H. Zhang F. Yuan N. J. Cao C. Fu H. Xie X.. (2017). “Beyond the words: Predicting user personality from heterogeneous information,” in Proceedings of the Tenth ACM International Conference on Web Search and Data Mining, pp. 305–314.

[B56] Witten I. H. Frank E. Hall M. A. Pal C. J. (2005). Data Mining: Practical Machine Learning Tools and Techniques (2nd ed). San Francisco: Morgan Kaufmann.

[B57] Worth N. C. Book A. S. (2014). Personality and behavior in a massively multiplayer online role-playing game. Comput. Hum. Behav. 38, 322–330. 10.1016/j.chb.2014.06.009

[B58] Worth N. C. Book A. S. (2015). Dimensions of video game behavior and their relationships with personality. Comput. Hum. Behav. 50, 132–140. 10.1016/j.chb.2015.03.05621780935

[B59] Wu F. Mulfinger E. Alexander I. I. I. L Sinclair A. McCloy R.. (2021). Individual differences at play: an investigation into measuring Big Five personality facets with game-based assessments. Int. J. Select. Assess. 21, 12360. 10.1111/ijsa.12360

[B60] Yee N. Ducheneaut N. Nelson L. Likarish P. (2011a). Introverted elves andamp; conscientious gnomes: the expression of personality in world of warcraft. Proceed. SIGCHI Conferen. Hum. Fact. Comput. Syst. 11, 753–762. 10.1145/1978942.1979052

[B61] Yee N. Harris H. Jabon M. Bailenson J. N. (2011b). The expression of personality in virtual worlds. Soc. Psychologic. Personal. Sci. 2, 5–12. 10.1177/194855061037905626799886

