# Supplementary material for: Predicting Risk Propensity Through Player Behavior in DOTA 2: A Cross-Sectional Study
Source: Front Psychol. 2022 Apr 29;13:827008. doi: 10.3389/fpsyg.2022.827008 (PMC9099285; doi:10.3389/fpsyg.2022.827008)
Supplement: Supplementary file 1 [file Table_1.pdf]

Supplementary Table 1. Description for selected features

Features selected	Description
rune pickups,	total number of runes picked up by the player
skewness of gold per min,	skewness of the gold earned by the player per min
mean of xp per min,	mean of the xp earned by the player per min
standard deviation of enemy creep kills per min,	standard deviation of the number of enemy creep killed by the player per min
number of attacking items purchasing,	total number of attacking items purchased by the player
number of comprehensive items purchasing,	total number of comprehensive items purchased by the player
times of items using,	total times of items used by the player
skewness of sentry ward planting,	skewness of the sentry ward planted by the player per min
number of necronomicon summoned units kills,	total number of necronomicon summoned units killed by the player
kurtosis of heroes kills per min,	kurtosis of heroes killed by the player per min
maximum hero hit,	maximum hero hit of the player
ratio of abilities cast on self,	number of abilities cast on self by the player / total number of abilities cast by the player
ratio of action type 9,	number of action type 9 / total number of actions
ratio of action type 10,	number of action type 10 / total number of actions
ratio of action type 11,	number of action type 11 / total number of actions
ratio of action type 13,	number of action type 13 / total number of actions
ratio of action type 14,	number of action type 14 / total number of actions
ratio of action type 16,	number of action type 16 / total number of actions
ratio of action type 20,	number of action type 20 / total number of actions
ratio of action type 23,	number of action type 23 / total number of actions
ratio of action type 26,	number of action type 26 / total number of actions
ratio of action type 32,	number of action type 32 / total number of actions

ratio of action type 36,	number of action type 36 / total number of actions
ratio of action type 38,	number of action type 38 / total number of actions
ratio of damage dealt by player,	total damage dealt by the player / total damage dealt by the team
ratio of damage dealt to creep,	total damage dealt to creep by the player / total damage dealt by the player
ratio of damage taken from creep,	total damage dealt taken from creep by the player / total damage taken by the player
mean of observer ward planting per min,	mean of observer ward planted by the player per min
standard deviation of sentry ward planting per min	standard deviation of sentry ward planted by the player per min
**mean of deaths in recent matches,**	average number of deaths in recent matches
**mean of xp per min in recent matches,**	average xp earned by the player per min in recent matches
**mean of tower damage in recent matches,**	average tower damaged by the player in recent matches
**total number of deaths,**	total number of deaths of the player in past matches
**KDA,**	(Kills + Deaths)/Assists
**total number of denies,**	total number of denies from the player in past matches
**lane efficiency pct,**	/
**total stun duration,**	total stun duration of the player in past matches
**total number of comebacks,**	total number of comebacks of the player in past matches
**loss**	/

Note. Features in ***bold*** *were historical statistic features crawled from Opendota. We have not figured out the meaning of lane efficiency pct and loss yet, but these two features could be crawled from Opendota directly without calculation.*
