# Supplementary material for: Predicting Risk Propensity Through Player Behavior in DOTA 2: A Cross-Sectional Study
Source: Front Psychol. 2022 Apr 29;13:827008. doi: 10.3389/fpsyg.2022.827008 (PMC9099285; doi:10.3389/fpsyg.2022.827008)
Supplement: Supplementary file 2 [file Image_1.pdf]

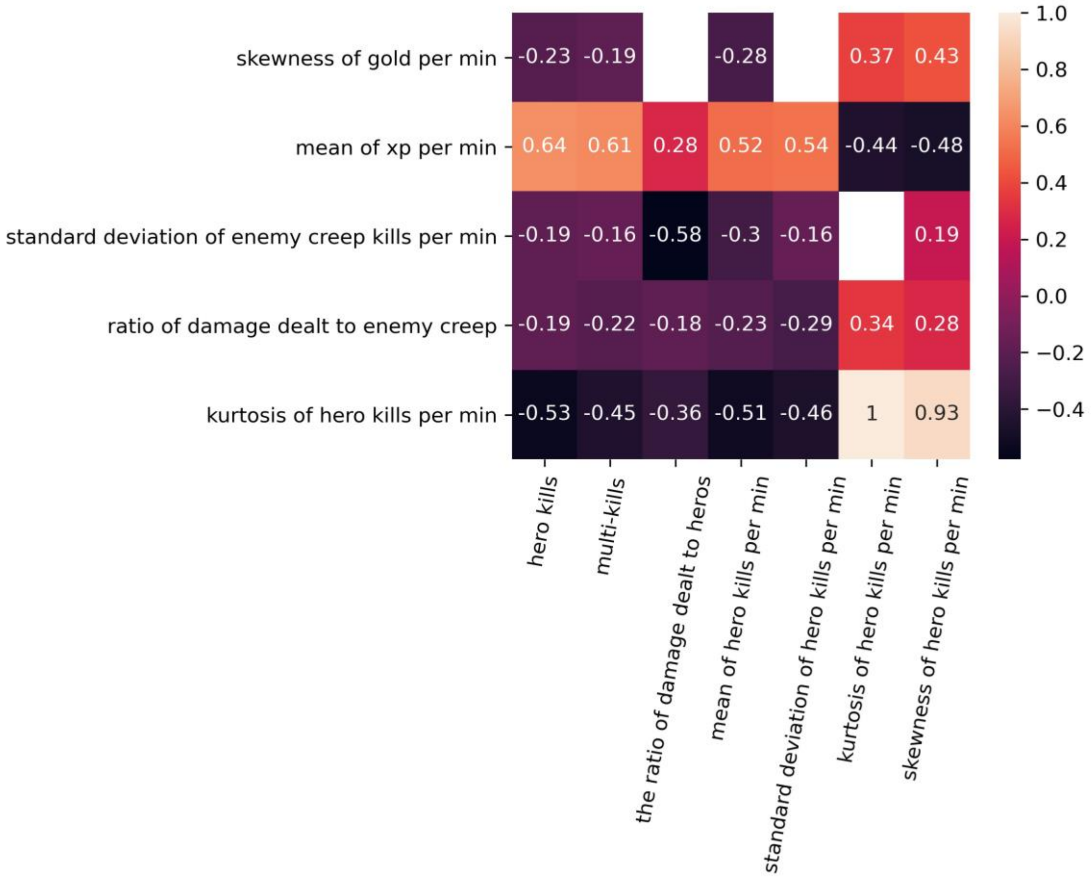

Supplementary Figure 1. Heatmap of the correlation coefficients between the features in the first category and hero killing behavior.
